# Nanoconjugation and Encapsulation Strategies for Improving Drug Delivery and Therapeutic Efficacy of Poorly Water-Soluble Drugs

**DOI:** 10.3390/pharmaceutics11070325

**Published:** 2019-07-10

**Authors:** Thao T. D. Tran, Phuong H. L. Tran

**Affiliations:** 1 Department for Management of Science and Technology Development, Ton Duc Thang University, Ho Chi Minh City, Vietnam; trantruongdinhthao@tdt.edu.vn; 2 Faculty of Pharmacy, Ton Duc Thang University, Ho Chi Minh City, Vietnam; 3 School of Medicine, Deakin University, Geelong 3216, Australia

**Keywords:** nanoconjugate, nanotechnology, poorly water-soluble drugs, theranostic, drug delivery, biomedical applications

## Abstract

Nanoconjugations have been demonstrated to be a dominant strategy for drug delivery and biomedical applications. In this review, we intend to describe several strategies for drug formulation, especially to improve the bioavailability of poorly water-soluble molecules for future application in the therapy of numerous diseases. The context of current studies will give readers an overview of the conjugation strategies for fabricating nanoparticles, which have expanded from conjugated materials to the surface conjugation of nanovehicles. Moreover, nanoconjugates for theranostics are also discussed and highlighted. Overall, these state-of-the-art conjugation methods and these techniques and applications for nanoparticulate systems of poorly water-soluble drugs will inspire scientists to explore and discover more productive techniques and methodologies for drug development.

## 1. Introduction

New drugs have been studied and developed rapidly worldwide. Unfortunately, these drugs may be limited in their clinical applications due to their poor solubility, adverse effects, or even toxicity. Over 70% of drugs on the current market, as well as recently discovered drugs, have been reported to be poorly water soluble [1,2,3,4,5]. These drugs require extensive research to improve their bioavailability due to low absorption or non-targeted delivery. Therefore, a number of strategies, such as solid dispersions, emulsions, prodrugs, and nanoparticles, have been investigated to improve the therapeutic index of poorly water-soluble drugs [6,7,8,9,10,11]. Nanotechnology applications in medicine have grown enormously, attracting researchers worldwide. Because of their small size and high surface area, nanosized drug particles have achieved encouraging outcomes in terms of improved drug solubility and bioavailability [12]. In addition, available surface modifications using nanotechnology can be applied in nano drug delivery systems for targeting drugs to specific sites, such as cancer tumours, for the development of targeted therapeutics and diagnostics [13].

**Figure 1:**
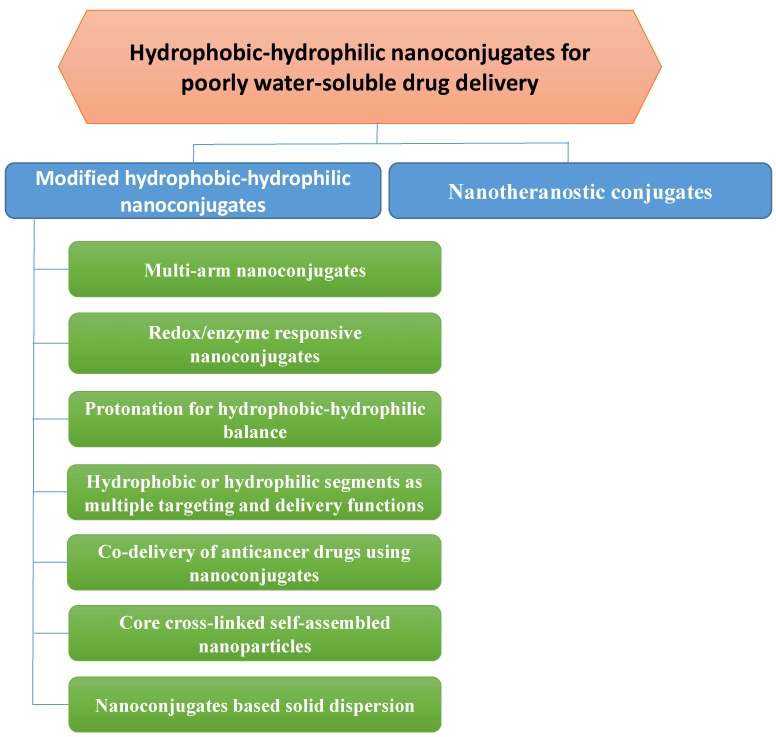
Illustration of nanoconjugation for improving drug delivery and therapeutic efficacy of poorly water-soluble drugs.

Although the use of hydrophilic polymers in solid dispersion, that is, the dispersion of a drug molecule in a carrier, has been established for improving drug dissolution and solubility since 1960 [14,15,16,17,18,19,20], hydrophilic–hydrophobic polymer nanoconjugates and hydrophobic drug-hydrophilic polymer nanoconjugates have been studied for loading drugs into nanoparticles for the same purposes in the past few decades of nanotechnological development [21,22,23]. The conjugation can commonly induce the formation of self-assembled polymeric amphiphiles, i.e., the exposure of this structure to aqueous environments results in self-assembled nanoparticles with hydrophobic segments in the inner core and hydrophilic segments towards the aqueous environment [9,24,25]. Consequently, the size of poorly water-soluble drugs is reduced to nanosize, leading to enhanced dissolution. Crystalline drug structure and drug-polymer interactions will be noted in addition to certain recent studies, as these factors may facilitate drug dissolution to improve bioavailability. With regard to theranostics systems for poorly water-soluble drugs, numerous nanoconjugate studies have been performed in cancer research [26,27]. In addition to the preferential accumulation of drug nanosized particles in tumours due to the leaky and porous structure of the tumoural blood vessels, nanoparticles may prolong the half-life of the drug in the blood circulation and specifically target a tumour by their surface decorations. However, conventional conjugations between hydrophilic molecules and hydrophobic molecules often encounter limitations such as low drug solubility, low drug loading, single drug delivery, large particle size, and short half-life, which may lead to unpredictable treatment efficacy. This review summarizes the current conjugation strategies for nanoparticulate formulations, suggesting efficient solutions to overcome these limitations and introducing specific applications of poorly water-soluble drugs (Figure 1).

## 2. General Hydrophobic-Hydrophilic Nanoconjugates for Poorly Water-Soluble Drug Delivery

**Figure 2:**
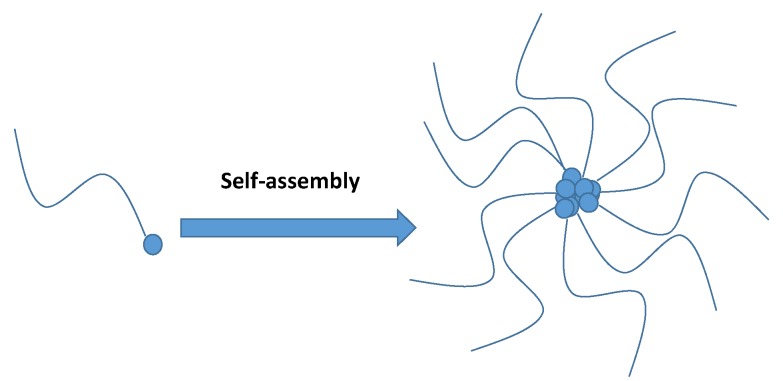
Illustration of self-assembled nanoparticles from hydrophobic–hydrophilic nanoconjugates. The blue dot represents the hydrophobic association.

Since the first reported study in 1984 introducing the formation of nanosized polymeric self-assemblies with potential as hydrophobic drug solubilizers [28], amphiphilic polymers have attracted the attention of researchers, especially for studies of nanomedicine in anticancer therapy [29,30]. Typically, conjugation strategies based on multiple interactions such as hydrogen bonding, host–guest interaction, hydrophobic interaction, and electrostatic interaction [31] have been proposed for the attachment of hydrophilic and hydrophobic segments to create an amphiphilic polymer, which is then commonly self-assembled into nanoparticles in an aqueous environment by molecular associations between hydrophobic segments (Figure 2) [32,33,34]. In other words, a hydrophobic segment can significantly affect the formation, drug encapsulation, drug interactions, and stability of nanoconjugates [35]. A poorly water-soluble drug can be loaded into hydrophobic moieties to improve its solubility, increase circulation time, target the tumour environment, and/or prevent drug degradation. In this review, we do not include the general information previously reported in recent prominent review articles on self-assembled nanoparticles such as polymer types, spacers, concentrations, and self-assembly mechanisms [36,37,38,39,40,41,42,43,44,45]. Instead, we focus only on the latest strategies, particularly on how the sophisticated conjugate is modified to improve the capacity of conventional hydrophilic–hydrophobic nanoconjugates to deliver poorly water-soluble drugs.

## 3. Modified Hydrophobic-Hydrophilic Nanoconjugates for the Delivery of Poorly Water-Soluble Drugs 

The extensive biomedical applications of nanoparticles arise from their small particle size. Their size enables nanoparticles to accumulate preferentially in tumour sites since tumour blood vessels are generally more heterogeneous in distribution, larger, and more permeable than normal blood vessels. The increased vascular permeability coupled with the impaired lymphatic drainage in rapidly growing tumours allows an enhanced permeability and retention (EPR) effect of the nanoparticles in the tumour. Thus, nanoparticles have been designed and engineered to have sizes and structures that are pertinent to biomedical applications such as targeted drug delivery, biomedical imaging, hyperthermia, and biosensing. However, the small size of nanoparticles might have incidental disadvantages in the context of unexpected drug solubility, particularly drug loading. Therefore, a failure of high drug loading or the achievement of enhanced drug solubility may significantly affect the therapeutic index or toxicity. A summary of works performed to improve those problems using nanoconjuagtes of poorly water-soluble drugs is presented in Table 1.

### 3.1. Multi-Arm Nanoconjugates for Poorly Water-Soluble Drug Delivery

Multi-arm nanoconjugates have recently been investigated to overcome these limitations [61,62,63,64]. For example, in a study of Sapra’s group on developing SN38 (the active metabolite of camptothecin), multi-arm polyethylene glycol was designed to conjugate to the model drug to enhance solubility [46,47]. This proposed delivery system has been demonstrated to confer significant preclinical therapeutic improvement and a longer half-life. More recently, natural pectin was decorated on polyethylene glycol with eight arms, which self-assembled into nanoparticles that were capable of improving drug solubility and controlling drug release [48]. This study showed that the optimal particle size (approximately 90 nm) could deliver two insoluble anticancer drugs, ursolic acid and hydrooxycampothecin, with efficient cellular uptake and cell cytotoxicity [48]. The in vivo test also indicated a higher survival rate in tumour-bearing mice administered the nanoparticles than in those administered the free drugs [48]. The presence of multiple arms on the conjugate structure was hence proposed as a strategy to achieve improved drug encapsulation efficiency, increased drug solubility, and suitable particle size for enhancing drug bioavailability. However, large numbers of conjugate arms may result in large aggregates due to the increased size and complexity of the structure. Therefore, the formulation approach of these multi-arm nanoconjugates should be designed appropriately.

### 3.2. Redox/Enzyme Responsive Nanoconjugates

To modulate drug release in the tumour microenvironment, redox/enzyme responsive linkage can be inserted into the hydrophobic–hydrophilic conjugate structure. An example of this type of conjugate is the disulfide linkage for redox-responsive drug delivery, which is cleaved by a high intracellular glutathione concentration [65,66,67]. Specifically, in the work of Liu et al. [49], cystamine was conjugated between deoxycholic acid (the hydrophobic segment) and chondroitin sulfate (the hydrophilic portion) for the delivery of docetaxel to treat melanoma. This system was designed as a dual-responsive drug release trigger because chondroitin sulfate was degraded by hyaluronidase-1 in addition to the redox-sensitive cystamine (with disulfide linkage) [49]. Generally, the use of responsive parts in nanoconjugates represents a promising drug delivery strategy with the advantage of facilitating drug release by an on/off switch in the desired environment [49,50]. Nevertheless, a surplus of the responsive part may affect the hydrophobic–hydrophilic balance, which must be optimized for nanoparticle self-assembly and maximum stability [68].

### 3.3. Protonation for Hydrophobic-Hydrophilic Balance

In addition to hydrophobic–hydrophilic parity, other factors, such as electrostatic and van der Waals interactions, play crucial roles in promoting the formation of self-assembled nanoparticles. For instance, excessive protonation or deprotonation within individual nanoconjugates results in electrostatic repulsion and weakened attractive forces. Dey et al. demonstrated the role of proton balance in the structure of chitosan for self-assembled nanoparticles [69]. Their study indicated that the partial protonation and partial deprotonation of chitosan could aid the self-assembly of nanoparticles for various applications ranging from wound-healing to gene delivery [69].

### 3.4. Hydrophobic or Hydrophilic Segments as Multiple Targeting and Delivery Functions

Simple hydrophobic–hydrophilic nanoconjugates commonly have limited ability to target tumour sites. Moreover, successful cancer treatment is determined by the ability of the therapeutic to eradicate the tumour while affecting as few healthy cells as possible. Therefore, the nanoconjugates can be actively targeted to tumours for receptor-mediated uptake by specifically recognizing and binding target tissues or cells via a surface-attached specific ligand, such as a “vector” molecule [70,71,72,73]. Interestingly, the hydrophobic core of conjugate-forming nanoparticles can also be used for synergistic targeting effects (in addition to the common functions of hydrophobic segments in self-assembly and the loading of poorly water-soluble drugs) to simplify the multi-step fabrication of the self-assembled nanoparticles [51]. For example, glycyrrhetinic acid, which is a hydrophobic targeting ligand for hepatocytes, was successfully conjugated with hyaluronic acid (as a targeting ligand on the surface of the nanoparticles) to deliver doxorubicin [51,74,75,76]. This study also noted that a conjugate’s biological function may be affected by the binding site and should be considered in polymer conjugate designs [51].

In addition to the conjugation between hydrophobic and hydrophilic segments, self-assembled nanoparticle preparation strategies may use poorly water-soluble drugs as hydrophobic segments in the core-shell structure. This approach can lead to highly stable nanoparticles and high drug loading. For example, Taxol has been proposed to conjugate to a cell-penetrating peptide to yield a high drug loading of 26.4% [52]. This supramolecular nanosphere formation (~130 nm) could also be used as a carrier to deliver doxorubicin. Liu et al. [53] found that cytarabine could be loaded up to 63% into stable self-assembled spherical nanoparticles using this strategy. Currently, the highest loading efficiency is 89.5%, found in a study of a paclitaxel and succinic acid conjugate forming self-assembled nanofibres [54]. In another study, in addition to enhancing oral bioavailability, capsaicin was successfully synthesized and formed self-assembled nanoparticles to reduce mucosa irritation [77].

A hydrophilic therapeutic agent was also used as a carrier, leading to the discovery of its dual functions in nanoconjugates. An example of such a dual-function material is fucoidan, which possesses the properties of a hydrophilic carrier and has also been demonstrated to be a potential anticancer agent [78,79,80,81,82,83]. Fucoidan was conjugated to oleic acid for the loading of paclitaxel and curcumin to maximize efficacy [55]. While the fucoidan and paclitaxel conjugate showed a preference for being released in a physiological environment, the fucoidan and curcumin conjugate showed improved drug release in a tumour environment [55]. For further theranostic development, fucoidan and oleic acid were functionalized on iron oxide nanoparticles [56]. This research showed that fucoidan may stabilize iron oxide nanoparticles as well as deliver poorly water-soluble drugs [56].

### 3.5. Co-Delivery of Anticancer Drugs using Nanoconjugates

**Figure 3:**
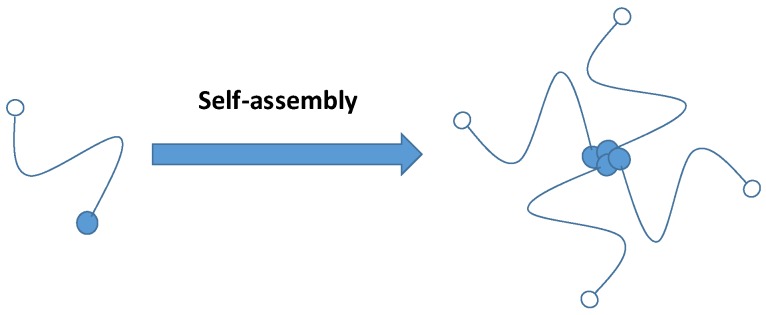
Illustration of drug co-delivery (blue and white dots) using a nanoconjugate through a spacer [57].

Combination therapy of anticancer drugs has shown potential in therapy due to multiple-mechanism actions, especially for resistant tumours, enabling the reduction of individual dosages and resulting in a synergistic effect and reduced toxicity [57,84,85]. Typically, one hydrophobic drug and one hydrophilic drug are combined and formulated with a hydrophilic carrier (also known as a spacer) for hydrophilic–hydrophobic balance in the structure (Figure 3). For example, docetaxel and gemcitabine were conjugated to polyethylene glycol, demonstrating that their therapeutic efficacy was significantly higher than that of individual drugs [57]. Similarly, Jain et al. bioconjugated gemcitabine and curcumin, likewise demonstrating that the conjugate is more effective than physically combined two drugs or a single drug [84]. Furthermore, Nam et al. [86] developed and compared fucoidan conjugated to curcumin and paclitaxel via an ester linkage. This system showed the dual delivery of two anticancer drugs, hydrophilic (fucoidan) and hydrophobic (curcumin, or paclitaxel) [86]. The curcumin–fucoidan conjugate released more drug in the acidic environment than the conjugate of paclitaxel and fucoidan [86].

Although the strategy of using a spacer to link hydrophobic drugs and hydrophilic drugs yielded impressive therapeutic outcomes, spacer properties such as length, type, and linkage site greatly influence in vitro and in vivo efficacy, which must be taken into account during synthesis [87]. Therefore, direct drug–drug conjugation via a biodegradable bond has been discussed, and researchers have developed self-assembled nanoparticles without using carriers. In such a case, one hydrophobic drug and one hydrophilic drug could self-assemble, combining their own hydrophilic and hydrophobic segments, and form nanostructures with high drug loading, high reproducibility and the potential to improve poor drug solubility [88]. This strategy was used by Huang et al., who conjugated irinotecan (hydrophilic) to chlorambucil (hydrophobic) to achieve excellent anticancer activity [58]. A floxuridine and bendamustine conjugate was also found to overcome multidrug resistance using this strategy [89].

### 3.6. Core Crosslinked Self-Assembled Nanoparticles

**Figure 4:**
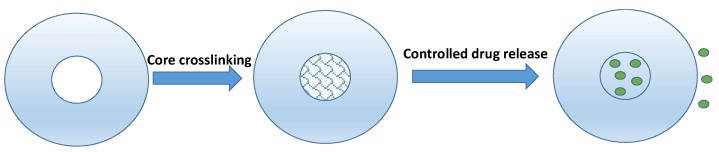
Illustration of core-crosslinked nanoconjugate for controlled drug release [92]. The green dots represent the drug molecules.

In an effort to control drug release, a core modification in self-assembled nanoparticles could be considered in addition to stimuli-responsive surface modifications, which have been widely reported [90,91]. The crosslinking degree was reported to have a strong effect on drug release in acidic environments [92]. Specifically, the core was crosslinked between furan rings and a crosslinker, resulting in a 10% or 20% degree of crosslinking to remain stable at high pH [92] (Figure 4). The core-crosslinked hyaluronic acid micelle demonstrated a high loading efficiency of a poorly water-soluble drug (>80%) [93]. The combination of lutetium-177-labelled core-crosslinked polymeric micelles and nanoparticles of cyclopamine synergistically delayed tumour growth in chemoradiation therapy [94]. In another recent study, docetaxel-loaded reduction-responsive core-crosslinked hyaluronic acid-b-poly(trimethylene carbonate-co-dithiolane trimethylene carbonate) micelles showed four-fold stronger tumour accumulation compared to free docetaxel [95].

### 3.7. Nanoconjugate-Based Solid Dispersion

**Figure 5:**
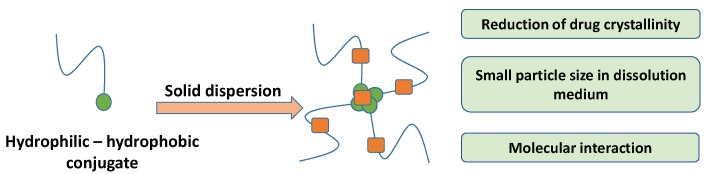
Illustration of the use of a hydrophilic–hydrophobic conjugate as a carrier in solid dispersion [59]. The drug molecules (orange dots) are dispersed between hydrophilic parts (blue strings) and hydrophobic parts (green dots).

Solid dispersion is a technique in which poorly water-soluble drugs are dispersed in hydrophilic carriers [20,96,97]. Although hydrophilic carriers can prevent drug recrystallization, drug crystals cannot always be transformed into amorphous forms due to the drug’s structure or polymer type. Ngo et al. suggested using hydrophilic–hydrophobic blends to overcome this limitation [98]. Furthermore, Dinh et al. developed a hydrophilic–hydrophobic conjugate using a carrier in solid dispersion to improve drug bioavailability [59] (Figure 5). This conjugate was demonstrated to be a potential carrier because it can induce a molecular interaction, reducing drug particles and changing drug crystals in solid dispersion [59,60].

## 4. Nanotheranostic Conjugates

Amphiphilic nanoconjugates have been investigated for concomitant therapy and diagnostics to take full advantages of nanoconjugates as single nanomaterials [99,100]. Generally, the imaging agent is incorporated into the hydrophobic core or on the hydrophobic segment for image monitoring. This incorporation would be a simple encapsulation in a conjugate or in complex chemical reactions with a conjugate. Some examples of recent strategies using amphiphilic nanoconjugates as multifunctional nanoparticles are as follows (Table 2):

Gadolinium is known for its efficient loading in amphiphiles to combine drug release with enhanced magnetic resonance signals [101]. In a study on an enzyme-sensitive biodegradable conjugate for treating breast cancer with paclitaxel, gadolinium was chelated to the conjugate by reacting it with GdCl_3_·6H_2_O in the dark for 24 h [102]. The in vivo magnetic resonance imaging in this study showed significant contrast enhancement and prolonged accumulation in a tumour [102]. In addition, a fluorescence study demonstrated the efficient accumulation of a cyanine 5.5-labelled nanoconjugate in a tumour [102].

In another study, the fluorochrome Rhodamine 6G was bonded to an amphiphile via reversible addition-fragmentation chain transfer (RAFT) polymerization for tumour fluorescence imaging detection [103,104]. Interestingly, the fluorescence intensity of Rhodamine 6G is pH dependent. As the nanoconjugates accumulated in the tumour’s acidic environment, the fluorescence intensity increased to enable cancer cell detection [103,104].

Chlorin e6 has been reported as an NIR fluorescence imaging dye and can be used in photodynamic therapy in biomedical applications [105,106,107,108]. Recently, the coupling of chlorin e6 was to hyaluronic acid via adipic dihydrazide was proposed for dual-modal imaging and phototherapy [109]. This study indicated that a chlorin e6-labelled nanoconjugate enhanced fluorescence and photoacoustic imaging by releasing chlorin e6 in a tumour [109]. Moreover, the accumulation of the nanoconjugate containing chlorin e6 demonstrated effective photodynamic therapy [91,109].

## 5. Future Prospects of Nanoconjugation for Poorly Water-Soluble Drugs

Harnessing the potential of nanoconjugation for poorly water-soluble drugs would be an effective approach to overcome barriers to the clinical translation of these drugs. However, despite extensive studies on the development of novel nanoconjugate systems with determined structures, the challenges of how to maximize targeting activity and therapeutic efficacy and minimize unwanted side effects remain. Burst drug release, incomplete drug dissolution, drug loading efficiency, and drug resistance all present difficult tasks that must be addressed and solved during the manufacture of these synthesized materials. Additional ongoing challenges include obtaining homogenous structures, achieving reproducible batch-to-batch synthesis, particularly of complex nanoconjugates, decreasing the time consumption of the synthesis, and scaling up product quality control. Nevertheless, a smart design of nanoconjugates in the first stage of a study, including the selection of materials, a rational design approach, formulations, a synthesis approach and efficient characterization techniques, would drive experimental studies to successful outcomes for translational applications.

## 6. Conclusions

A wide range of nanoconjugates has been developed to improve the bioavailability of poorly water-soluble drugs. Significant efforts in recent studies have demonstrated improved effects of nanoconjugates on drug solubility, particle size, and drug co-delivery, and delivery. Specifically, the strategy of direct conjugation between hydrophilic and hydrophobic drugs could facilitate the generation of nanoconjugates without the use of carriers, resulting in the advantages of high drug loading and limited batch variation. Further investigations of these efficient drug delivery systems should focus on clinical translations. Furthermore, optimization and scale-up procedures should also be attempted and addressed.

## Figures and Tables

**Table 1:** Examples of studies on nanoconjuagtes of poorly water-soluble drugs for therapeutics.

Poorly Water-Soluble Drugs	Approaches	Key Results	References
SN38 (the active metabolite of camptothecin)	Multi-arm	Enhanced drug solubility. Significant preclinical therapeutic improvement and a longer half-life of the drug.	[46,47]
Ursolic acid and 10-hydroxycamptothecin		Effective cellular uptake. Higher survival rate of tumour-bearing mice.	[48]
Docetaxel	Redox/enzyme responsive	Triggering dual-responsive drug release. Facilitating drug release by an on/off switch in the desired environment.	[49,50]
Doxorubicin	Multiple targeting	Synergistic targeting effect.	[51]
Paclitaxel Doxorubicin Cytarabine	Poorly water-soluble drugs as hydrophobic segments in the core-shell structure	High drug loading.	[52,53,54]
Fucoidan Paclitaxel Curcumin	The combination use of a hydrophilic therapeutic agent	Dual functions.	[55,56]
Docetaxel	Hydrophobic drug-spacer-hydrophilic drug conjugates	Co-delivery of anticancer drugs.	[57]
Chlorambucil	Hydrophobic drug-hydrophilic drug conjugates	Excellent anticancer activity.	[58]
Isradipine Prednisolone	Solid dispersion	Improve drug bioavailability.	[59,60]

**Table 2:** Example of studies on nanotheranostic conjugates of poorly water-soluble drugs.

Poorly Water-Soluble Drugs	Imaging Agent	Key Results	References
Paclitaxel	Gadolinium	Significant contrast enhancement. Prolonged accumulation in a tumour.	[102]
Doxorubicin	Rhodamine 6G	Increased fluorescence intensity for cancer cell detection.	[103,104]
Doxorubicin	Chlorin e6	Enhanced fluorescence and photoacoustic imaging. Effective photodynamic therapy.	[91,109]
